# Adherence to SARS-CoV-2 Vaccination Recommendations among Patients with Substance Use Disorders: A Cross-Sectional Study in Rome, Italy

**DOI:** 10.3390/vaccines11091434

**Published:** 2023-08-30

**Authors:** Francesco Mondera, Vincenzo Cammalleri, Francesca Maria Forestiero, Federica Turatto, Giovanni F. M. Direnzo, Anna Napoli, Francesca Pirelli, Nirinalisera Razafimpanana, Ettore Rossi, Valentina Baccolini, Lilia Cinti, Carolina Marzuillo, Massimo Barra, Guido Antonelli, Aldo Badiani, Paolo Villari

**Affiliations:** 1 Department of Public Health and Infectious Diseases, Sapienza University of Rome, 00185 Rome, Italy; vincenzo.cammalleri@uniroma1.it (V.C.); francescamaria.forestiero@uniroma1.it (F.M.F.); federica.turatto@uniroma1.it (F.T.); valentina.baccolini@uniroma1.it (V.B.); carolina.marzuillo@uniroma1.it (C.M.); paolo.villari@uniroma1.it (P.V.); 2 Villa Maraini Foundation, 00151 Rome, Italy; gfmdirenzo@gmail.com (G.F.M.D.); ettorerossi@villamaraini.it (E.R.); massimo.barra@cri.it (M.B.); aldo.badiani@uniroma1.it (A.B.); 3 Department of Molecular Medicine, Sapienza University of Rome, 00185 Rome, Italy; anna.napoli@uniroma1.it (A.N.); lilia.cinti@uniroma1.it (L.C.); guido.antonelli@uniroma1.it (G.A.); 4 Department of Physiology and Pharmacology, Sapienza University of Rome, 00185 Rome, Italy; francesca.pirelli@uniroma1.it (F.P.); nirinalisera@gmail.com (N.R.)

**Keywords:** vaccines, vaccination coverage, health disparities, COVID-19, substance use disorders, vaccine hesitancy

## Abstract

Adherence to vaccination recommendations is a challenge for national immunization programs. We quantified adherence to COVID-19 vaccination recommendations in people with substance use disorders (SUDs) attending an outpatient addiction center in Rome, Italy; we investigated the determinants of adherence, and also analyzed patient risk perception and compliance with preventive measures. A multivariable logistic regression model identified predictors of adherence to vaccination recommendations, with statistical validity tested by estimating adjusted odds ratios (aORs) and 95% confidence intervals (CIs). From December 2021 to January 2022, 200 SUD patients completed a questionnaire, 80% of whom reported being vaccinated against SARS-CoV-2 (minimum one dose). Negative predictors of vaccine uptake included being non-Italian (aOR: 0.36, 95% CI: 0.13–0.97), having coexisting comorbidities (aOR: 0.35, 95% CI: 0.13–0.95), and previous use of heroin (aOR: 0.24, 95% CI: 0.08–0.71). No difference was found for cocaine use, demographic characteristics, previous COVID-19 infection, methadone therapy, or compliance with preventive measures. Major reasons for non-adherence to vaccination recommendations were fear of side effects, insufficient recognition of the importance of vaccination, bureaucratic issues, and lack of trust in the authorities. Given their vulnerability, additional efforts are needed to facilitate access to vaccination for people with SUDs, and to limit disinformation around vaccines..

## 1. Introduction

The COVID-19 pandemic has challenged healthcare systems worldwide, causing tens of thousands of deaths and consistent economic damage in many countries [1,2]. Thanks to mass vaccination campaigns, however, a progressive reduction in mortality rates has been observed since January 2021, confirming the efficacy, quality and safety of newly developed vaccines [3,4]. Nevertheless, the COVID-19 vaccination campaign was hindered by the concomitant infodemic, with misinformation and disinformation about the vaccines being especially prominent on social networks [5,6]. This has fueled vaccine hesitancy [7,8], a phenomenon defined by the Strategic Advisory Group of Experts on Immunization as a “delay in acceptance or refusal of vaccination despite availability of vaccination services” [9], which is considered to be one of the major threats to global health over the last decade [10]. Vaccination hesitancy is a function of a variety of individual, social, cultural, and economic factors [9,11]. 

A growing number of studies have investigated vaccine hesitancy and adherence to COVID-19 vaccination recommendations in the general population and in specific subgroups [12,13,14]. Unfortunately, there are still limited data on hard-to-reach populations. In particular, little is known about COVID-19 vaccination rates in people with substance use disorders (SUDs) [15]. This population has suffered disproportionally during the COVID-19 pandemic. In many cases, addiction service providers were obliged to reduce or terminate their in-person activities altogether, while sometimes offering alternative telemedicine services [16,17]. This has been detrimental to the fragility and isolation of their clients [18,19]. Moreover, there is evidence that people with SUDs are less likely than the general population to adhere to vaccination programs against influenza, diphtheria, pneumococcal infection, and hepatitis B [20,21].

Therefore, the aims of this study were (i) to quantify adherence to anti-SARS-CoV-2 vaccination recommendations in a sample of people with SUDs, and to identify the predictors of adherence; and (ii) to analyze COVID-19 risk perception in the SUD population, and their adherence to the recommended precautions. This information might provide important information to policymakers for promoting immunization against COVID-19 and, potentially, other important infectious diseases.

## 2. Materials and Methods

### 2.1. Study Design and Setting

This cross-sectional study was conducted between December 2021 and January 2022 in Rome, Italy, in the outpatient drug treatment center of the Villa Maraini Foundation, part of the National Agency of the Italian Red Cross for pathological addictions. Founded in 1976, Villa Maraini provides several services for the treatment and rehabilitation of patients suffering from drug use, alcohol abuse and gambling addiction. 

The sample size needed in the survey (i.e., 200 participants) was estimated using the Cochran formula for small populations [Table S1], with the following parameters as inputs: population size, 1645 (number of patients at Villa Maraini); proportion of the sample with the expected outcome (i.e., having received at least one dose of anti-SARS-CoV-2 vaccine), 82% (based on previous projections on the Italian population); margin of error, ±5.0%; confidence level, 95.0%. We used a convenience sample of patients attending the Villa Maraini Foundation. After receiving care, patients were asked to take part in the study, and informed consent was obtained from each one. No incentive was offered. Then, we tested every participant with a rapid oropharyngeal swab for the presence of SARS-CoV-2 infection, and we administered a questionnaire on demographics, health status, previous COVID-19 infection, substances used and related behaviors, preventive measures adopted during the pandemic, adherence to SARS-CoV-2 vaccination schedules, and COVID-19 risk perception. Ethical approval for this study was obtained from the Ethics Committee of Sapienza University of Rome (Prot. 0697/2021). 

### 2.2. Questionnaire

The questionnaire was derived from a literature review and was developed in collaboration with the personnel of the Villa Maraini Foundation. It consisted of 25 questions grouped into 4 sections [Table S2]. It was administered by a member of the research team and took approximately 20 min to fill out.

The first section aimed to collect general information: age, gender, nationality, weight, educational level, work status, accommodation status (type and number of cohabitants).

The second section focused on the respondents’ COVID-19 experience and pathological and toxicological history. We investigated any previously known COVID-19 infection or influenza-like symptoms of illness. Then, we asked whether respondents had ever used heroin, cocaine, street methadone or cannabis during the pandemic, alone or with others. In addition, we asked them if they were in therapy with methadone, and whether they had any coexisting comorbidity, and about their smoking habits (non-smoker, former smoker or current smoker).

The third section explored COVID-19 vaccination status. We asked whether they had received at least one dose of COVID-19 vaccination, the vaccine type received (if any), number of doses (if any), and to report the main reasons for vaccination non-compliance.

The last section investigated the participants’ self-reported adherence to recommended precautionary measures during the pandemic, and their perception of the risks around COVID-19. Specifically, we asked respondents to report the most common type of mask used, and to rate from 0 (never) to 10 (always) how frequently they usually wore a community or surgical mask indoors with non-cohabiting people, or any face mask outdoors when recommended. Participants were also surveyed about how frequently (on a scale from 0 to 10) they performed hand hygiene procedures when recommended and respected physical distancing during outdoor activities. Lastly, we asked them to rate from 0 (none) to 10 (always) how worried they were for their own health, for the health of their loved ones, and for the health of other people regarding COVID-19. 

### 2.3. Statistical Analysis

Descriptive statistics were obtained using the median and interquartile range or mean and standard deviation for continuous variables, and proportions for dichotomous and categorical variables. For the purposes of this analysis, participants were considered to be Italian or of other nationalities. Work status was grouped as either stable and in work or unstable/unemployed. Regarding housing, we categorized our sample as unstable (homeless or with precarious accommodation), living alone, or living with others.

For those who had received at least one dose of COVID-19 vaccination, their vaccination cycle was categorized as incomplete (if they had received only one dose out of a two-dose vaccine schedule), complete (i.e., two doses out of a two-dose vaccine schedule, or one dose out of a one-dose vaccine schedule), or with booster (if they had received a further dose after a primary cycle). Reasons for refusal to accept vaccination were grouped into five categories: “considering it unnecessary”, “having bureaucratic problems accessing vaccination centers”, “being afraid of the side effects”, “not trusting the authorities”, and “others”.

Patients that reported receiving at least one dose of vaccine were considered adherent to anti-SARS-CoV-2 vaccination recommendations. For the univariable analyses, the Wilcoxon rank sum test was used to compare continuous variables between vaccination-adherent and non-adherent patients, whereas Pearson’s chi-squared or Fisher’s exact test was used for dichotomous and categorical variables. A multivariable logistic regression model was built to identify predictors of adherence to anti-SARS-CoV-2 vaccination recommendations. Variables were included in the model based on expert opinion. Multicollinearity was checked using a variance inflation factor of 5 as threshold. The Hosmer and Lemeshow test was used to evaluate the goodness of fit of the model. The final model consisted of the following variables: gender (dichotomous), age (continuous), nationality (dichotomous), educational level (dichotomous), coexisting comorbidities (dichotomous), housing (categorical), previous COVID-19 infection (dichotomous), heroin use (dichotomous), cocaine use (dichotomous), being in therapy with methadone (dichotomous), type of daily mask used (categorical), adherence to indoor mask wearing (continuous), adherence to outdoor mask wearing (continuous), performing hand washing (continuous) and maintaining physical distancing (continuous). Adjusted odds ratios (aORs) and 95% confidence intervals (CIs) were calculated.

All analyses were performed using Stata (StataCorp LLC, 4905 Lakeway Drive, CollegeStation, TX 322, USA), version 17.0. A two-sided *p*-value < 0.05 was considered statistically significant.

## 3. Results

Between December 2021 and January 2022, 200 patients with SUDs attending the outpatient center of Villa Maraini foundation were surveyed. They were mainly males (84.5%) and were aged 43.9 ± 10.6 years on average (Table 1). Most respondents were Italian (72.0%). Half of the sample had an academic level of at least a high school degree (50.0%) and were of normal weight (51.2%), whereas a slightly greater proportion (62.0%) reported being unemployed or having casual or occasional work. Regarding housing, 115 participants (57.5%) reported sharing an apartment with other people; 59 patients (29.5%) lived alone, and 26 subjects (13.0%) had unstable accommodation arrangements. In a univariable analysis of the data, no meaningful difference was found between adherent and non-adherent patients for most variables, with two exceptions only: compared to people of other nationalities, a higher proportion of Italian participants were adherent to COVID-19 vaccination recommendations (86.8% vs. 62.5%, *p* < 0.001); similarly, compared to the other weight classes, obese participants had a higher rate of vaccination compliance (*p* = 0.002).

By contrast, comparing only those who had receive one dose of SARS-CoV-2 vaccine (i.e., incomplete vaccination schedule) to the entire sample, we did not detect any difference [Table S3]. 

Considering the pathological and toxicological history (Table 2), a minority of patients (7.5%) reported a previously known COVID-19 infection, while more than half of our sample reported having had influenza-like symptoms (55.5%) during the pandemic (Table 2). We also found that the majority of our patients had used heroin (60.5%) in the previous two years, around half of them had used cocaine (51.5%), 39.0% had used cannabis, and 78 participants (43.1%) had used substances together with other people. A large proportion of our population was still in therapy with methadone (87.4%) and were smokers (94.5%) at the time of the survey; approximately one in every two participants (54.0%) reported having at least one comorbidity, with the most represented one being a psychiatric/neurological disorder [Table S4]. As for their vaccination status, most of our patients (N = 160, 80.0%) reported having received at least one dose of COVID-19 vaccine. The most common vaccines administered were Pfizer-BioNTech, with 58 vaccinated individuals, and Moderna, with 49 individuals (36.9% and 31.2%, respectively). A total of 103 patients had already completed the primary cycle, and 38 participants (24.2%) reported having received a booster dose. Among unvaccinated individuals, the most frequent reasons given for not being vaccinated were considering it unnecessary and being afraid of the side effects (27.5% and 25.0%, respectively), followed by having had bureaucratic problems accessing vaccination centers, and lack of trust in the authorities (17.5% each). In the univariable analysis, heroin users had lower rates of vaccination adherence (72.7% vs. 91.1%, *p* = 0.001), as did those still in therapy with methadone (77.6% vs. 96.0%, *p* = 0.032). No other meaningful difference was found.

Regarding preventive measures for COVID-19 (Table 3), more than half the sample used community masks only (57.0%), while use of a filtering facepiece (FFP) FFP2 or FFP3 was next most frequent (27.5%) (Table 3). As for self-reported compliance, the use of a mask indoors and frequency of hand washing scored the highest (average 7.5 out of 10 for both). In contrast, the use of a mask outdoors returned the lowest score (average 4.4), whereas maintenance of physical distancing scored an average or 6.4. As for COVID-19 risk perception, our patients reported the highest concern for infection of their loved ones (average 6.9), followed by concern for other people (average 5.8) and themselves (average 4.3). Comparing vaccinated and unvaccinated patients, the only meaningful difference was found in the type of mask used, with the highest proportion of patients vaccinated with at least one dose being in the FFP2 or FFP3 mask group (90.9%).

In the multivariable analysis (Table 4), being of other nationalities showed a negative association with adherence to vaccination recommendations (aOR: 0.36, 95% CI: 0.13–0.97) (Table 4). Similarly, having a comorbidity and having used heroin during the pandemic was associated with a reduction in vaccine uptake (aOR: 0.35, 95% CI: 0.13–0.95 and aOR: 0.24, 95% CI: 0.08–0.71, respectively). No difference was found in relation to cocaine use, demographic characteristics (such as sex, age, work status, accommodation status, and educational level), previous COVID-19 infection, being in therapy with methadone, or any other behavioral variable.

## 4. Discussion

In this survey, we investigated adherence to COVID-19 vaccination recommendations in a specific subgroup of people, i.e., a sample of patients with SUDs attending an outpatient drug treatment center in Rome, Italy, and we found that between December 2021 and January 2022, 80% of the participants had received at least one dose of vaccine. Despite this value being slightly lower than the 85% officially reported by the National Institute of Health in the same period for the general Italian population in the age range 40–59 years [22,23], an age category comparable with that of our sample, it was higher than that found in Australia [24] and Mexico [25] among people who injected drugs (41% and 39%, respectively). Hence, these data suggest that the vaccination policies adopted by the Italian government, such as the implementation of mandatory vaccinations for specific subgroups of workers or people aged 50 or above, together with the introduction of a green pass for everyone who wanted to enter public locations, may have contributed to the relatively high degree of vaccine uptake in the SUD subgroup, directly or indirectly [26]. It is worth mentioning the different times at which the surveys were conducted, with our study enrolling participants one year after the COVID-19 vaccine became available, i.e., from two to four months after the Australian and Mexican investigations; however, it is unlikely that the discrepancy between studies is attributable to this factor alone. However, we found that an appreciable number of Italian patients with SUDs still considered the vaccination to be unnecessary, had concerns about side effects, or claimed bureaucratic issues as obstacles in getting vaccinated. Therefore, since these patients may be at high risk of severe outcomes following COVID-19 infection [27], tailored efforts should be made to reach this population subgroup, such as offering the opportunity to be vaccinated while receiving care at the outpatient center, a scenario that has already occurred in Spain [15], where providing brief medical advice to people with opioid disorders and referring them to vaccination clinic sites made it possible for approximately 70% of these people to be fully vaccinated. 

As for predictors of adherence to vaccination recommendations, in contrast with other studies in which socioeconomic disparities were reported in the general population of the United States [28], the United Kingdom [29], and Italy [30], we did not detect any difference in our sample according to sex, age, work status, or educational level. By contrast, as already reported [31,32], we found an association within the univariable analysis between higher weight and vaccination rate, but more studies are needed to confirm this finding and make hypotheses. On the other hand, despite in principle having the same access to vaccination as Italians, foreign participants seemed to have suffered from a greater difficulty in accessing vaccination centers, as previously noted [30]. This finding, which also remained significant upon multivariable analysis, could be due to a combination of factors (including distrust in government policies or fear of deportation for immigrants with an illegal status), but it makes clear the importance of providing people with timely and reliable information about the procedures for getting vaccinated and the benefits of vaccination, limiting disinformation as much as possible [33]. Countering disinformation and fake news may be critical in contrasting any potential concern about the side effects of vaccination, a factor that may explain why people in relatively poor health also had a lower vaccination rate. This consideration may also apply to heroin users, who in our study were the only type of drug user that was less likely to obtain vaccination, even though we did not delve into the characteristics of their drug dependence. An alternative explanation could be that people abusing opioids may wish, for various reasons, to limit contact with health professionals, including those offering vaccination [24]. However, since this contrasts with what was reported for Australian and American drug users [24,34], a deeper understanding of the relationship between the type of drug used and vaccination adherence is warranted.

The perceived risk of contracting COVID-19 is among the most important factors that influence both attitudes and behavior [26,35]. In our study, participants showed greater concern that their loved ones or other people might become infected than that they themselves would contract the virus, suggesting that participants did not feel at particularly high risk of infection. In addition, we found that self-reported adherence to the guidelines was slightly lower than that reported in another study that used a similar methodology, but targeted university students [12]; again, the different times at which the two surveys were conducted might explain this. As aforementioned, our study took place one year after the vaccine was released, when evidence on the effectiveness of vaccination in preventing hospitalization and death had started to accumulate [36]. Moreover, in our sample, the highest compliance was reached in relation to mask wearing indoors and hand washing, two of the non-pharmaceutical interventions most effective in containing the spread of the virus. However, although we failed to find a relationship between compliance with any particular guideline and vaccine uptake, using FFP2 or FFP3 as the only mask almost reached statistical significance, a behavior that could result from considerations of both personal and societal health benefits [37]. Indeed, this may show how one of the key messages of many communication campaigns across the world, i.e., the importance of implementing non-pharmaceutical interventions to prevent the spread of the virus and thereby protect others, has been carefully considered by this subgroup, showing that they have understood why the recommended measures are useful for them and their community [38].

This study has some strengths and limitations. Firstly, the cross-sectional design hindered the opportunity to draw causal conclusions between vaccination adherence and the associated factors. Secondly, all answers were self-reported; therefore, social desirability bias cannot be excluded. Nevertheless, despite our sample being relatively small, it may represent the target population (i.e., patients with SUDs served at the Villa Maraini Foundation) fairly well. In addition, it should be noted that while some addiction service providers were obliged to offer telemedicine services or to deliver several therapy sessions in a single day (both of which had a negative impact on the fragility and isolation of their patients) [18,19], this was not the case for the Villa Maraini Foundation, which maintained the same level of service for the entire duration of the pandemic, allowing us to conduct one of the few studies in Italy that investigated vaccination adherence in people with SUDs under treatment in an outpatient center. Lastly, we were able to analyze patients’ risk perceptions and adherence to recommended precautions in relation to vaccination compliance, providing data that may support policymakers in developing effective communication strategies for the promotion of vaccine uptake in this specific subgroup. 

## 5. Conclusions

One year after the start of the vaccination campaign, around one in five participants in our sample of Italian SUD patients reported not being vaccinated against SARS-CoV-2. A few factors were negative predictors of vaccine uptake, such as being of other nationalities and having coexisting comorbidities, with several patients reporting fear of side effects, lack of recognition of the importance of getting vaccinated, bureaucratic issues, and lack of trust in the authorities as major reasons for non-adherence. Therefore, since patients with SUDs are usually hard-to-reach people, additional efforts to increase their awareness and engagement, facilitate their access to vaccination centers, and limit disinformation regarding the vaccines should be made.

## Figures and Tables

**Table 1:** General characteristics of the participants (N = 200). Results are expressed as mean (standard deviation, SD), median (interquartile range, IQR), or frequency (percentage).

	Total	(%)	Adherence to COVID-19 Vaccination		*p*-Value
	N		N	(%)	
Sex					0.283
Male	169	(84.5)	133	(78.7)	
Female	31	(15.5)	27	(87.1)	
Age, years					
Mean (SD)	43.9	(10.6)	44.3	(9.1)	0.206
Median (IQR)	44	(35.5–52)	46	(35–53)	
Nationality					<0.001
Italian	144	(72.0)	125	(86.8)	
Others	56	(28.0)	35	(62.5)	
Educational level					0.724
Middle school diploma or below	100	(50.0)	79	(79.0)	
High school diploma or above	100	(50.0)	81	(81.0)	
BMI category (N = 198)					0.002
Underweight (BMI < 18.5)	24	(12.3)	14	(58.3)	
Normal weight (BMI between 18.5 and 24.9)	102	(51.2)	85	(86.8)	
Overweight (BMI between 25.0 and 29.9)	52	(26.3)	39	(75.0)	
Obese (BMI > 30)	20	(10.2)	20	(100.0)	
Work status					0.244
Occasional work or unemployed	124	(62.0)	96	(77.4)	
Stable work	76	(38.0)	64	(84.2)	
Housing					0.133
Unstable	26	(13.0)	17	(65.4)	
Living alone	59	(29.5)	49	(83.1)	
Living with others	115	(57.5)	94	(81.7)	
Number of cohabitants					0.486
None	80	(52.5)	64	(80.0)	
One or two	95	(47.5)	75	(79.0)	
At least three	25	(12.5)	21	(84.0)	
Living with someone aged 65 years old or above					0.642
No	165	(82.5)	131	(79.4)	
Yes	35	(17.5)	29	(82.8)	

BMI: body mass index (kg/height in meter^2^). COVID-19: coronavirus disease 2019. Wilcoxon test for continuous variables, chi^2^ test or Fisher’s test for categorical variables.

**Table 2:** Pathological, toxicological, and COVID-19 vaccination history of the participants (N = 200). Results are expressed as frequency (percentage).

	Total	(%)	Adherence to COVID-19 Vaccination		*p*-Value
	N		N	(%)	
Previous COVID-19 infection					0.506
No	185	(92.5)	149	(80.5)	
Yes	15	(7.5)	11	(73.3)	
Previous influenza-like illness symptoms					0.064
No	89	(44.5)	66	(74.2)	
Yes	111	(55.5)	94	(84.7)	
Use of heroin					0.001
No	79	(39.5)	72	(91.1)	
Yes	121	(60.5)	88	(72.7)	
Use of cocaine					0.056
No	97	(48.5)	83	(85.6)	
Yes	103	(51.5)	77	(74.8)	
Use of street methadone					0.141
No	174	(87.0)	142	(81.6)	
Yes	26	(13.0)	18	(69.2)	
Use of cannabis					0.346
No	122	(61.0)	95	(77.9)	
Yes	78	(39.0)	65	(83.3)	
Use of substances with others (N = 168)					0.649
No	99	(56.9)	79	(79.8)	
Yes	75	(43.1)	58	(77.3)	
In therapy with methadone (N = 199)					0.032
No	25	(12.6)	24	(96.0)	
Yes	174	(87.4)	135	(77.6)	
Coexisting comorbidity					0.119
No	92	(46.0)	78	(84.8)	
Yes	108	(54.0)	82	(75.9)	
Smoking habit					0.374
Non-smoker or former smoker	11	(5.5)	11	(100.0)	
Current smoker	189	(94.5)	149	(78.8)	
Received at least one dose of anti-COVID-19 vaccine					
No	40	(20.0)			
Yes	160	(80.0)			
COVID-19 vaccine type received (N = 157)					
Pfizer-BioNTech	58	(36.9)			
AstraZeneca	11	(7.0)			
Moderna	49	(31.2)			
Johnson & Johnson	13	(8.3)			
Heterologous vaccination ^a^	26	(16.6)			
Vaccination cycle (N = 157)					
Incomplete (only one dose out of two)	16	(10.2)			
Complete	103	(65.6)			
Booster dose	38	(24.2)			
Reasons for vaccination non-adherence (N = 38)					
Considering it unnecessary	11	(27.5)			
Not trusting the authorities	7	(17.5)			
Having bureaucratic problems accessing vaccination centers	7	(17.5)			
Being afraid of the side effects	10	(25.0)			
Others	3	(7.5)			

COVID-19: coronavirus disease 2019. Chi^2^ test or Fisher’s test for categorical variables. ^a^ Vaccination cycle completed with two different vaccines in any combination, or booster dose vaccine different from primary cycle vaccine.

**Table 3:** Participants’ self-reported adherence to COVID-19 prevention measures (N = 200). Results are expressed as mean (standard deviation, SD), median (interquartile range, IQR), or frequency (percentage).

	Total	(%)	Adherence to COVID-19 Vaccination		*p*-Value
	N		N	(%)	
Type of mask used					0.038
Surgical mask only	114	(57.0)	89	(78.1)	
FFP2 or FFP3 mask only	55	(27.5)	50	(90.9)	
Fabric mask only	20	(10.0)	14	(70.0)	
More than one type	11	(5.5)	7	(63.6)	
Frequency of mask use indoors with non-cohabiting people ^a^					
Mean (SD)	7.5	(3.2)	7.5	(3.1)	0.631
Median (IQR)	9	(6–10)	8.5	(6–10)	
Frequency of mask use outdoors ^a^					
Mean (SD)	4.4	(3.9)	4.4	(3.8)	0.518
Median (IQR)	4	(0–8)	4	(0–8)	
Frequency of hand washing ^a^					
Mean (SD)	7.5	(2.8)	7.3	(2.9)	0.206
Median (IQR)	8	(6–10)	8	(5–10)	
Frequency of maintaining interpersonal distance (N = 198) ^a^					
Mean (SD)	6.4	(3.4)	6.2	(3.4)	0.134
Median (IQR)	8	(4–9)	7	(4–9)	
Concern for own health in case of COVID-19 disease (N = 199) ^a^					
Mean (SD)	4.3	(3.8)	4.4	(3.8)	0.294
Median (IQR)	4	(0–8)	5	(0–10)	
Concern for the health of loved ones in case of COVID-19 disease (N = 198) ^a^					
Mean (SD)	6.9	(3.8)	7.2	(3.6)	0.108
Median (IQR)	9	(4–10)	9	(5–10)	
Concern for the health of other people in case of COVID-19 disease (N = 199) ^a^					
Mean (SD)	5.8	(3.7)	6	(3.5)	0.138
Median (IQR)	7	(2–9)	7	(4–9)	

COVID-19: coronavirus disease 2019. FFP: filtering facepiece. Wilcoxon test for continuous variables, Fisher’s exact test for categorical variables. ^a^ Responses were given on a scale from 0 (never/not at all) to 10 (always/a lot).

**Table 4:** Multivariable logistic regression model for adherence to COVID-19 vaccination.

	aOR (95%CI)	*p*-Value
Sex (female)	1.73 (0.41–7.26)	0.449
Age, years	1.04 (0.99–1.09)	0.098
Nationality (others)	0.36 (0.13–0.97)	0.045
Educational level (high school diploma or higher)	0.86 (0.36–2.03)	0.731
Coexisting comorbidity (yes)	0.35 (0.13–0.95)	0.040
Work status (stable work)	1.18 (0.45–3.15)	0.732
Accommodation status		
Unstable	Ref.	
Living alone	1.90 (0.49–7.15)	0.361
Living with others	1.71 (0.50–5.88)	0.388
Previous COVID-19 infection (yes)	0.61 (0.14–2.69)	0.518
Use of cocaine (yes)	0.79 (0.31–1.97)	0.618
Use of heroin (yes)	0.24 (0.08–0.71)	0.011
In therapy with methadone (yes)	0.64 (0.64–6.50)	0.714
Type of mask used		
More types	Ref.	
Fabric mask only	2.00 (0.30–13.22)	0.474
Surgical mask only	2.91 (0.58–14.52)	0.192
FFP2 or FFP3 mask only	6.12 (0.94–39.40)	0.057
Frequency of mask use indoors with non-cohabiting people	0.94 (0.80–1.11)	0.523
Frequency of maintaining interpersonal distance	0.90 (0.77–1.06)	0.249
Frequency of hand washing	0.90 (0.73–1.08)	0.264
Frequency of mask use outdoors	1.07 (0.94–1.23)	0.278

aOR: adjusted odds ratio. CI: confidence interval. COVID-19: coronavirus disease 2019. FFP: filtering facepiece.

## Data Availability

The data presented in this study are available on request from the corresponding author.

## Supplementary Materials

The following supporting information can be downloaded at: https://www.mdpi.com/article/10.3390/vaccines11091434/s1, Table S1: Formula used in the sample size (s) calculation. Table S2: Survey questionnaire. Table S3: General characteristics of the participants that reported an incomplete vaccination cycle at the time of the survey (N = 16). Table S4: Self-reported comorbidities of the participants (N = 200).
