# Supplementary material for: Adherence to SARS-CoV-2 Vaccination Recommendations among Patients with Substance Use Disorders: A Cross-Sectional Study in Rome, Italy
Source: Vaccines (Basel). 2023 Aug 30;11(9):1434. doi: 10.3390/vaccines11091434 (PMC10538200; doi:10.3390/vaccines11091434)
Supplement: Supplementary file 1 [file vaccines-11-01434-s001.zip › vaccines-2559219-supplementary.pdf]

Table S1. Formula used in the sample size (s) calculation.

$s = (Z^2 p\ (1\text{-}p) / m^2) / [1 + ((Z^2 p\ (1\text{-}p) / m^2) - 1) / P]$	
Z	z-value (1.96)
p	proportion of population with the expected outcome (0.82)
m	margin of error (0.05)
P	total population (1645).

Table S2. Survey questionnaire.

Date ____/______/_____________ Place _______________

1. Weight _______	2. Height _______
3. Sex ☐ M ☐ F	4. Age ___________
5. Nationality ☐ Italian ☐ Other (specify) ________________	

6. Highest degree?
☐ None
☐ Primary school
☐ Secondary school
☐ High school
☐ Bachelor's degree

7. Work condition?
☐ Unemployed
☐ Occasional employement
☐ Part-time
☐ Full-time
☐ Student
☐ Retired
☐ Freelance
☐ Other

8. Housing?

☐ Alone	Go to question 9	
☐ Dormitory		
☐ Unstable (e.g., a friend's house)		
☐ Homeless		
☐ Sharing an apartment	8.1. How many people?	(number)___________

	8.2. Over 65 years old?	☐ Yes (number)____<br>☐ No
	8.3. Under 18 years old?	☐ Yes (number)____<br>☐ No

9. Which of the following ones is applicable to you?

☐ I contracted COVID-19	9.1 Where the diagnosis was made?<br>☐ during hospitalization/in the hospital<br>☐ by local health unit swab
☐ I have been in contact with a person with COVID-19	Go to question 10
☐ I had symptoms but did not run further tests	
☐ None of the above	

10. Have you ever been tested before for COVID-19?

☐ Yes, it was negative	10.1 If yes, reccomended by who?<br>☐ Hospital staff<br>☐ General practitioner<br>☐ Self<br>☐ Employer<br>☐ Villa Maraini Foundation<br>☐ Other _________________
☐ Yes, it was positive	
☐ No	Go to question 11

11. Have you ever done a serological test for COVID-19 antibodies?

☐ Yes, it was negative	11.1 If yes, recommended by who?<br>☐ Hospital staff<br>☐ General practitioner<br>☐ Self<br>☐ Employer<br>☐ Villa Maraini Foundation<br>☐ Other _________________
☐ Yes, it was positive	
☐ No	Go to question 12

12. Have you ever experienced the following symptoms since the beginning of the pandemic? (multiple options can be selected)

☐ Fever
☐ Cough
☐ Sore throat
☐ Loss of taste
☐ Loss of smell
☐ Cold
☐ Breathlessness
☐ Asthenia
☐ Conjunctiitis
☐ Unusual muscle aches
☐ Diarrhea
☐ None →

12.1 If you experienced one of the previous symptoms, what have you done?

☐ Nothing
☐ I consulted my general practitioner
☐ I called the ministry of health freephone
☐ I asked my friends and family for help
☐ I self-quarantined
☐ I went to the hospital
☐ I asked to be visited by Villa Maraini staff
☐ I took medication on my own

13.Have you been vaccinated against COVID-19?

☐ Yes, one dose<br>☐ Yes, two doses<br>☐ Yes, three doses	13.1 What vaccines?<br>☐ Pfizer/BionTech _____<br>☐ Moderna _____<br>☐ AstraZeneca _____<br>☐ Other _____<br>______________________________	13.2 When? *(as precise as possible)*<br>____________________<br>________
☐ No	Go to question 14	

14. Why? ________________________________

15. Have you ever been diagnosed with any chronic disease? *(Multiple options can be selected)*

☐ Hypertension
☐ Heart diseases
☐ COPD
☐ Asthma
☐ Diabetes
☐ Obesity
☐ HCV ☐ cured
☐ HBV
☐ HIV ☐ in therapy
☐ Psychiatric/neurological
☐ Other
____

16. Do you take medications routinely?

☐ Yes	16.1 What medications?<br>______________________________
☐ No	Go to question 17

17. Do you smoke?

☐ Yes	17.1 What do you smoke?<br>☐ Tobacco<br>☐ E-cig<br>☐ Htps	17.2 How often?<br>☐ Less than 1 times a week<br>☐ 2-3 times a week<br>☐ Daily	17.3 How many?<br>☐ Less than 5<br>☐ Between 5 and 9<br>☐ Between 10 and 14<br>☐ Between 15 and 2<br>☐ More than 20
☐ No	Go to question 18		

18. Did you take one or more of these substances during the pandemic period?

(*Way of intake: i.v.=intravenous, i.m.=intramuscolar, f=smoked, s=sniffed, os=oral; Frequency: daily/ more than 3 times a week/ during weekend/ once a week / 1-3 times a month/ less than 10 times a year/ 1 or 2 times a year)*

Substance	Way of intake	Frequency
Heroin		
Cocaine		
Street methadone		
Benzodiazepines		
Other psychiatric drugs		
Alcohol		
Cannabinoids		
Ketamine		
Ecstasy		
Other ________________________		

19. Do you take drugs alone? ☐ Alone
☐ With others

20.Do you share the tools to take the substance?

☐ Never
☐ Rarely
☐ Sometimes
☐ Often
☐ Always

21. Are you being treated with replacement therapies?

☐ Yes	21.1 What drugs?<br>☐ MTD 1%<br>☐ MTD 5%<br>☐ Levo MTD<br>☐ Buprenorfina	21.2 Last dose?<br>____________________	21.3 Schedule and frequency<br>☐ stable dose<br>☐ variable dose<br>☐ regular frequency<br>☐ irregular frequency
☐ No	Go to question 22		

22. From the onset of the pandemic have you ever had an overdose?

☐ Yes	22.1 How many times?<br>____________________	22.2 What happened?<br>☐ I went to the ER<br>☐ I have been assisted by Villa Maraini staff<br>☐ A relatives (or similar) gave me naloxone
☐ No	Go to question 23	

23. What type of mask do you use the most?

☐ FFP2 o FFP3
☐ Surgical mask
☐ Community mask (fabric or similar)

24. On a scale from 0 (never) to 10 (always), how often do you:

keep your mask indoor with non-cohabiting people?	0	1	2	3	4	5	6	7	8	9	10
keep you mask on outside?	0	1	2	3	4	5	6	7	8	9	10
wash your hands or disinfect them with a hydroalcoholic solution?	0	1	2	3	4	5	6	7	8	9	10
keep interpersonal distance?	0	1	2	3	4	5	6	7	8	9	10

25. On a scale from 0 (none) to 10 (extremely), how much are you concerned about:

yourself	0	1	2	3	4	5	6	7	8	9	10
your loved	0	1	2	3	4	5	6	7	8	9	10

ones											
other people	0	1	2	3	4	5	6	7	8	9	10

Table S3. General characteristics of the participants that reported an incomplete vaccination cycle at the time of the survey (N=16).

	Total		Incomplete vaccination cycle		*p*-value
	N	(%)	N	(%)	
Sex					0.999
Male	169	(84.5)	14	(8.3)	
Female	31	(15.5)	2	(6.5)	
Age, years					0.574
Mean (SD)	43.9	(10.6)	42.4	(12.3)	
Median (IQR)	44	(35.5-52)	42	(31-50)	
Nationality					0.077
Italian	144	(72.0)	8	(5.6)	
Non-Italian	56	(28.0)	8	(14.3)	
Educational level					0.724
Middle school diploma or below	100	(50.0)	6	(6..0)	
High school diploma or above	100	(50.0)	10	(10.0)	
BMI category (N=198)					0.433
Underweight (BMI <18.5)	24	(12.3)	2	(8.3)	
Normal weight (BMI between 18.5 and 24.9)	102	(51.2)	11	(10.8)	
Overweight (BMI between 25.0 and 29.9)	52	(26.3)	3	(5.8)	
Obese (BMI > 30)	20	(10.2)	0	(0.0)	
Work status					0.966
Occasional work or unemployed	124	(62.0)	10	(8.1)	
Stable work	76	(38.0)	6	(7.9)	
Housing					0.867
Unstable	26	(13.0)	1	(3.9)	
Living alone	59	(29.5)	5	(8.5)	
Living with others	115	(57.5)	10	(8.7)	
Number of cohabitants					
None	80	(52.5)	6	(7.5)	0.270
One or two	95	(47.5)	6	(6.3)	
At least three	25	(12.5)	4	(16.0)	
Living with someone aged 65 years old or above					0.999
No	165	(82.5)	14	(8.5)	
Yes	35	(17.5)	2	(6.7)	

BMI: body mass index (kg/height in meter^2). COVID-19: coronavirus disease 2019.
Wilcoxon test for continuous variables, chi^2 test or Fisher's test for categorical variables.

Table S4. Self-reported comorbidities of the participants (N=200).

	Total		Adherence to COVID-19 vaccination		
	N	(%)	N	(%)	*p*-value
Hypertension					0.578
No	179	(89.5)	144	(80.4)	
Yes	21	(10.5)	16	(76.2)	
Heart diseases					0.200
No	192	(96.0)	155	(80.7)	
Yes	8	(4.0)	5	(62.5)	
COPD					0.726
No	187	(93.5)	150	(80.2)	
Yes	13	(6.5)	10	(76.9)	
Asthma					0.026
No	187	(93.5)	153	(81.8)	
Yes	13	(6.5)	7	(53.8)	
Diabetes					0.999
No	194	(97.0)	155	(79.9)	
Yes	6	(3.0)	5	(83.3)	
HCV					0.292
No	135	(67.5)	110	(81.5)	
Yes	21	(10.5)	14	(66.7)	
Cured	44	(22.0)	36	(81.8)	
HBV					0.999
No	186	(93.0)	149	(80.1)	
Yes	14	(7.0)	11	(78.6)	
HIV					0.186
No	183	(91.5)	146	(79.8)	
Yes	4	(2.0)	2	(50.0)	
In therapy	13	(6.5)	12	(92.3)	
Psychiatric/neurological disorder					0.415
No	172	(86.0)	136	(79.1)	
Yes	28	(14.0)	24	(85.7)	
Other (e.g., cancer, CKD)					0.275
No	176	(88.0)	143	(81.2)	
Si	24	(12.0)	17	(70.8)	

COPD: Chronic Obstructive Pulmonary Disease. HCV: Hepatitis C Virus. HBV: Hepatitis B Virus. CKD: Chronic Kidney Disease.
